# Evaluation of Neuropathological Features in the SOD1-G93A Low Copy Number Transgenic Mouse Model of Amyotrophic Lateral Sclerosis

**DOI:** 10.3389/fnmol.2021.681868

**Published:** 2021-06-24

**Authors:** Agnes Molnar-Kasza, Barbara Hinteregger, Joerg Neddens, Roland Rabl, Stefanie Flunkert, Birgit Hutter-Paier

**Affiliations:** QPS Austria GmbH, Grambach, Austria

**Keywords:** neuroinflammation, muscle phenotype, spinal cord, survival rate, muscle strength, body weight

## Abstract

Amyotrophic lateral sclerosis (ALS) still depicts an incurable and devastating disease. Drug development efforts are mostly based on superoxide dismutase 1 gene (SOD1)-G93A mice that present a very strong and early phenotype, allowing only a short time window for intervention. An alternative mouse model is available, that is based on the same founder line but has a reduced SOD1-G93A copy number, resulting in a weaker and delayed phenotype. To be able to use these SOD1-G93A/low mice for drug testing, we performed a characterization of ALS-typical pathologies. All analyses were performed compared to non-transgenic (ntg) littermates of the same sex and age. *In vivo* analysis of SOD1-G93A/low mice was performed by weekly body weight measurements, analysis of the survival rate, and measurement of the muscle strength of 24–30 weeks old female and male SOD1-G93A/low mice. Immunofluorescent labeling of SOD1, glial fibrillary acidic protein (GFAP), and ionized calcium-binding adaptor molecule 1 (Iba1) protein was performed in the cervical, thoracic, and lumbar ventral horn of the spinal cord of 24–30 weeks old male and female SOD1-G93A/low mice. The musculus gastrocnemius of male SOD1-G93A/low mice was labeled with fluorophore-conjugated α-bungarotoxin and antibodies against phosphorylated neurofilaments. Fluorescent labeling was detected and quantified by macro-based image analysis. Although SOD1 protein levels were highly increased in both sexes and all age groups, levels strongly peaked in 30 weeks old male SOD1-G93A/low mice. Astrocytosis and activated microglia in the spinal cord ventral horn and phosphorylated neurofilaments in the motor unit of the musculus gastrocnemius progressively increased, while muscle strength progressively decreased in male SOD1-G93A/low mice. In female SOD1-G93A/low mice, only activated microglia increased progressively, while muscle strength was constantly reduced starting at 26 weeks. These differences result in a shorter survival time of male SOD1-G93A/low mice of about 3 weeks compared to female animals. The results suggest that male SOD1-G93A/low mice present a stronger pathology and are, therefore, better suitable to evaluate the efficacy of new drugs against ALS as most pathological features are developing progressively paralleled by a survival time that allows treatment to start before symptom onset.

## Introduction

After many years of research, amyotrophic lateral sclerosis (ALS) is still a devastating and incurable disease that usually manifests with a fast progression and a mean survival time of <5 years after diagnosis. Most common symptoms include progressive muscle weakness and wasting that eventually lead to respiratory failure [for review see Masrori and Van Damme (2020)]. Preclinical ALS research requires animal models that are based on the same genetic alterations as observed in patients and in parallel closely mimic the disease pathology. The most commonly used ALS animal model is the superoxide dismutase 1 gene (SOD1) transgenic mouse model with G93A mutation (Gurney et al., 1994). This model presents a very severe phenotype including motor deficits, SOD1 aggregations, loss of spinal motor neurons, and a mean survival rate of only 18.5 weeks (Gurney et al., 1994). Although this model shows many phenotypic and pathological symptoms that are comparable to patients with ALS, it even outplays the human pathology in some aspects. It was shown that SOD1-G93A mice develop a severe intracytoplasmic vacuolar pathology in the neuropil and motor neurons, while this pathology is barely observed in the brain of patients with ALS (Dal Canto and Gurney, 1997; Shibata, 2001). An alternative SOD1 mouse model for preclinical research is the SOD1-G93A mouse with a lower copy number. This mouse is based on the same founder mouse by Gurney et al. (1994) but has only about 10–12 copy numbers of the transgene (Dal Canto and Gurney, 1997; Spooren and Hengerer, 2000; Sasaki et al., 2004). Phenotypically, these SOD1-G93A/low mice present a later disease onset and a mean survival time of about 34–36 weeks (Osman et al., 2020). Although first changes in lumbar motor neurons of SOD1-G93A/low mice can be observed already at the age of 2 weeks (Pambo-Pambo et al., 2009), developing limb paralysis is more variable in these mice compared to the original model (Osman et al., 2020). Additionally, animals present dysphagia and associated tongue atrophy that are congruent with the hypoglossal nucleus pathology in patients with ALS (Osman et al., 2020). Spinal cord pathology of SOD1-G93A/low mice presents similar features compared to the original model, but the vacuolar pathology is minimal and thus closer mimicking the human disease (Dal Canto and Gurney, 1997; Shibata, 2001).

As the longer survival time of SOD1-G93A/low mice provides a longer margin for treatment studies, and the model closer mimics the human disease, we characterized the pathological features and the *in vivo* phenotype in 24, 27, and 30 weeks old animals. The results of this study will allow to improve the design of future treatment studies in SOD1-G93A/low mice. In the first step, we evaluated SOD1 levels and the region size of the cervical, thoracic, and lumbar ventral horn of the spinal cord. Afterward, the neuroinflammatory profile of these mice was evaluated by GFAP and Iba1 labeling of the same ventral horn regions to evaluate levels of astrocytosis and activated microglia. As SOD1-G93A/low mice are known to develop limb paralysis, we additionally analyzed skeletal muscle samples for pathological changes. Phenotypic analysis of SOD1-G93A/low mice included weekly analysis of the body weight, motor performance in the wire suspension test, and an analysis of the survival rate. To evaluate sex differences, most analyses were performed for female and male animals separately.

## Method

### Animals

All mice were purchased from Jackson Laboratories (#002300), USA, under a license agreement with the Northwestern University, USA and housed under identical conditions in individually ventilated cages on standardized rodent bedding (Rettenmaier®, Germany) in the Association for Assessment and Accreditation of Laboratory Animal Care (AAALAC)-accredited animal facility of QPS Austria GmbH. Cotton nestlets (Plexx®, The Netherlands) were provided as nesting material. The room temperature (RT) was kept at ~21°C and the relative humidity between 40 and 70%. Mice were housed under constant light-cycle (12 h light/dark). Dried pelleted standard rodent chow (Altromin®, Germany) and normal tap water were available to the animals *ad libitum*. Each animal was checked regularly for any clinical signs. Mice were housed in same-sex groups of up to four animals. Animals were marked by ear punch. The study was started with 36 female and 36 male hemizygous SOD1-G93A/low mice and 25 female and 25 male non-transgenic (ntg) littermates. After behavioral tests at the age of 24, 27, and 30 weeks, eight animals per group were sacrificed for histological analyses. Every 3 weeks, the group size thus shrank about eight animals per group. Actual animal numbers are given in the figure legends. Animal studies conformed to the Austrian guidelines for the care and use of laboratory animals (Tierversuchsgesetz 2012-TVG 2012, BGBl. I Nr. 114/2012). Animal experiments were approved by the Styrian government (Amt der Steiermärkischen Landesregierung, Abteilung 13 – Umwelt und Raumordnung Austria; ABT13-23611/2019).

SOD1-G93A/low mice [B6.SJL-Tg (SOD1^*^G93A)^dl^1Gur/J, # 002300] express a variant of the human SOD1 with glycine to alanine substitution at position 93 (G93A) on a C57BL/6 × SJL background. This strain has roughly 30% fewer copies of the transgene construct than the high copy number line Tg(SOD1^*^G93A)1Gur (Gurney et al., 1994).

### Body Weight

The body weight of all animals was weighed weekly using a standard scale.

### Wire Suspension Test

The wire suspension test assesses muscular strength. For this test, a wire cage lid was used with duct tape placed around the perimeter to prevent the mouse from walking off the edge. The animal was placed on the top of the cage lid. The lid was slightly shaken to force the mouse to grip the wires and the lid was then turned upside down. The lid was held at a height of ~50–60 cm above a soft underlay, high enough to prevent the mouse from jumping down but not high enough to cause harm in the event of a fall. The latency to fall off the wire was measured. A 300 s cut-off time was used, although a healthy mouse can hang upside down for several minutes.

### Survival Analysis

At the age of 24, 27, and 30 weeks, eight animals per group were sacrificed and four randomly chosen animals were processed for histological analysis. The remaining animals were further kept, and clinical signs were evaluated daily. Once an animal reached a defined humane endpoint, it was euthanized by cervical dislocation. Euthanasia criteria included a weight loss of more than 20%, cramps, paralysis, hunched posture, pain while seizing, self-induced trauma, strong changes in body temperature as measured rectally or respiration as evaluated by visual inspection, blood in urine or feces, loss of righting reflexes (from both sides within 15–30 s.), and tumor of >2 cm. Additionally, an accumulation of weaker symptoms of the above-described symptoms that summed up to be a severe phenotype was considered decisive for euthanasia. Animals with milder motor symptoms that were not able to reach the food grid received food on the ground and water in bottles with an extended lid.

### Tissue Sampling

All mice were anesthetized by i.p. injection of 600 mg/kg pentobarbital. Once animals were deeply anesthetized and reflexes were not measurable, animals were transcardially perfused with physiological (0.9%) saline, and the spinal column and the musculus gastrocnemius were dissected. Both tissues were immediately fixed in freshly prepared 4% paraformaldehyde in 0.1 M phosphate buffer (pH 7.4) for 2 h at 4°C. Subsequently, spinal cords were dissected from spinal columns and all tissues were cryo-protected in a 15% sucrose/phosphate-buffered saline (PBS) solution overnight at 4°C. On the next day, spinal cords were dissected using the overall size and shape of the spinal cord to determine boundaries of cervical, thoracic, and lumbar parts. However, we did not attempt to identify individual segments. The spinal cord parts were then arranged as tissue array in cryomolds (one cryomold per spinal cord), embedded in OCT, and then snap-frozen in liquid dry ice-cooled isopentane.

### Immunofluorescent Labeling of Human SOD1, GFAP, and Iba1

Spinal cords were cryosectioned on a cryotome (Leica) at 10 μm thickness. Three tissue array sections per animal, each tissue array section including two cervical sections, five thoracic sections, and two lumbar sections were air-dried for 45 min, postfixed for 10 min in 4% paraformaldehyde solution (#0335.3, Merck, Darmstadt, Germany) at RT and then washed three times in PBS (PO4-360000, Pan Biotech, Aidenbach, Germany). Afterward, antigen demasking was performed with 1x citrate buffer (AP-9003, Thermo Scientific, Waltham, Massachusetts, USA) for 15 min at 95°C. After 15 min of cooling the sections to RT, sections were treated with freshly prepared 1 mg/ml sodium borohydride/PBS solution for 4 min, washed three times in PBS again, and subsequently blocked with M.O.M. blocking reagent (Vector Laboratories, Burlingame, California, USA) in 0.1% TritonX-100/PBS for 60 min in a humid chamber at RT. Afterward, tissue sections were washed 3 × 5 min in PBS and incubated with primary antibodies [anti-SOD1 antibody, 1:100, (MM-0070-2P), Medimabs; anti-GFAP antibody, 1:1000, (Z0334), Dako, and anti-Iba1 antibody, 1:2000, (230045), Synaptic Systems], overnight at 4°C. The primary antibody binding was visualized by labeling with the corresponding fluorophore-conjugated secondary antibodies [1:500, anti-mouse IgG (H+L) DyLight 650, Thermo Fischer Scientific, SA5-10169; 1:500, anti-rabbit IgG (H+L), DyLight 488, Abcam, ab96919; 1:500, anti-guinea pig IgG (H+L), Cyanine Cy™3, Jackson ImmunoResearch, 706-165-148; 1:500, anti-goat IgG (H+L), Alexa Fluor 750, Abcam, ab175745] for 1 h at RT. After washing in PBS, cell nuclei were labeled using DAPI counterstaining (A1001, PanReac AppliChem GmbH, Darmstadt, Germany) and sections were washed in PBS and distilled water and covered with Moviol® (Sigma Aldrich, St. Louis, USA) and coverslips.

### Immunofluorescent Labeling of Acetylcholine Receptors, Phosphorylated Neurofilaments, and Protein Gene Product (PGP) 9.5 in Muscle Tissue

Muscles were longitudinally cryosectioned on a cryotome and three 10 μm thick sections were air-dried for 45 min, postfixed for 10 min with 4% paraformaldehyde solution (#0335.3, Merck, Darmstadt, Germany) at RT and then washed three times in PBS (PO4-360000, Pan Biotech, Aidenbach, Germany). Afterward, sections were treated with freshly prepared 1 mg/ml sodium borohydride/PBS solution for 4 min, washed three times in PBS, and subsequently blocked with M.O.M. blocking reagent (Vector Laboratories, Burlingame, California, USA) containing α-bungarotoxin, 1:1,000, [B35451], Thermo Fisher Scientific; in 0.1% TritonX-100/PBS for 60 min in a humid chamber at RT. Afterward, tissue sections were washed 3 × 5 min in PBS and incubated with primary antibodies anti-phosphorylated neurofilaments (H+M) antibody, 1:2,000, [SMI-31P], Covance, and anti-PGP 9.5 antibody, 1:2,000, [516-3340], Zytomed Systems, overnight at 4°C. The primary antibody binding was visualized by labeling with the corresponding fluorophore-conjugated secondary antibodies [1:500, anti-mouse IgG (H+L) DyLight 650, Thermo Fischer Scientific, SA5-10169 and 1:500, anti-rabbit IgG (H+L), Alexa Fluor 750, Abcam, ab175728] for 1 h at RT. After washing in PBS, cell nuclei were labeled using DAPI counterstaining (A1001, PanReac AppliChem GmbH, Darmstadt, Germany) and sections were washed in PBS followed by distilled water and covered with Moviol® (Sigma Aldrich, St. Louis, USA) and coverslips.

### Image Preparation and Analysis

Whole slide scans of labeled sections were recorded on a Zeiss automatic microscope AxioScan Z1 with high aperture lenses, equipped with a Zeiss Axiocam 506 mono and a Hitachi 3CCD HV-F202SCL camera and Zeiss ZEN 2.3 software. Image analysis was performed with Image-Pro 6 (Media Cybernetics, Silver Spring, Maryland, USA). Image analysis was performed as described previously (Etxeberria-Rekalde et al., 2020). Target areas (cervical, thoracic, lumbar ventral horn, and muscle tissue) were identified by drawing areas of interest (AOI). AOI in the spinal cord included both sides. The image analysis was macro-based and ran automatically so the results are operator-independent and fully reproducible. Raw data were edited in Microsoft Excel and transferred to GraphPad Prism for statistical analysis and preparation of graphs.

### Statistics

Data analysis was performed in GraphPad Prism™ 8.1 (GraphPad Software Inc., USA). Graphs show group means and SEM. The significance level was set at *p* < 0.05. Group means were compared using two-way ANOVA with a subsequent *post-hoc* test. The utilized statistical tests and exact sample numbers are mentioned in the figure legends.

Raw data of all analyses including group sizes of each week are provided in Supplementary Table 1.

## Results

### SOD1 Protein Expression and Brain Atrophy

**Figure 1:**
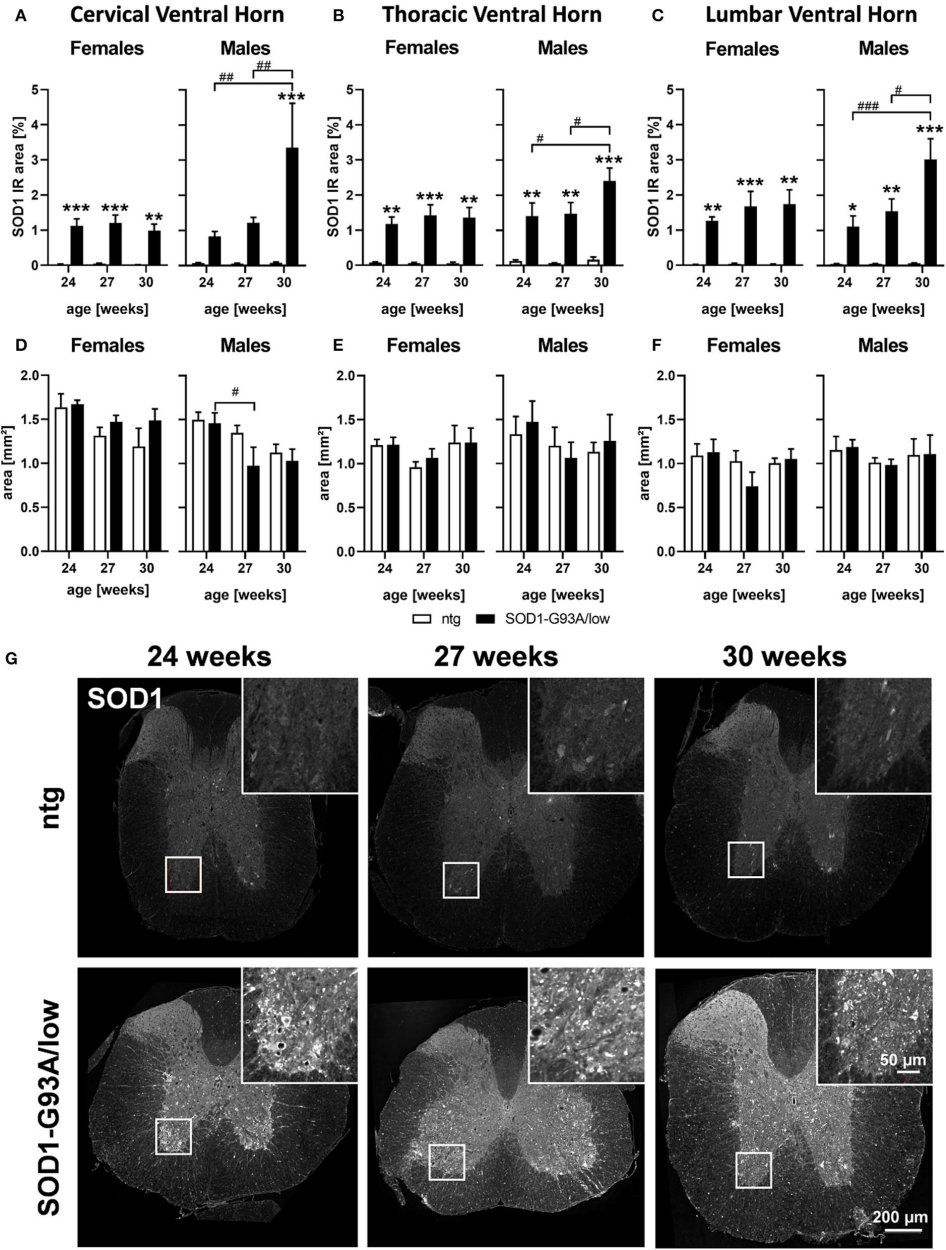
SOD1 protein expression and region size in the spinal cord of 24–30 weeks old SOD1-G93A/low mice. Female and male 24, 27, and 30 weeks old SOD1-G93A/low mice were evaluated for SOD1 expression **(A–C)** and ventral horn region size **(D–F)** in the cervical **(A,D)**, thoracic **(B,E)**, and lumbar **(C,F)** ventral horn compared to ntg littermates. Two-way ANOVA with Tukey's and Sidak's multiple comparison *post hoc* test; *n* = 4 per group. Mean + SEM. */^#^*p* < 0.05, **/^*##*^*p* < 0.01, ***/^*###*^*p* < 0.001. *Differences between genotypes; ^#^differences between age groups. **(G)** Representative images of SOD1 labeling in the thoracic spinal cord of 24, 27, and 30 weeks old male SOD1-G93A/low mice compared to ntg littermates.

Human SOD1 levels were evaluated in the spinal cord of 24, 27, and 30 weeks old female and male SOD1-G93A/low mice and ntg littermates. The SOD1 immunoreactive (IR) area in all three ventral horn regions of female SOD1-G93A/low mice was already highly increased at the age of 24 weeks and stayed stable with age (Figures 1A–C). Evaluation of male SOD1-G93A/low mice showed similar SOD1 IR area levels at the age of 24 and 27 weeks than female animals, but at the age of 30 weeks, an additional increase in SOD1 levels compared to younger male SOD1-G93A/low mice was measurable (Figures 1A–C). Particularly in the lumbar ventral horn, a progressive increase of SOD1 levels could be observed in male SOD1-G93A/low mice (Figure 1C).

Afterward, the brain region size of the cervical, thoracic, and the lumbar ventral horn was evaluated in female and male SOD1-G93A/low mice. Our results show that male SOD1-G93A/low mice presented a reduced region size of the cervical ventral horn at the age of 27 weeks compared to SOD1-G93A/low mice at the age of 24 weeks (Figure 1D). In female animals and other ventral horn areas no differences in the region size between genotypes and age groups could be observed (Figures 1D–F).

Representative images of SOD1 labeling in the thoracic spinal cord of SOD1-G93A/low mice and ntg littermates at the age of 24, 27, and 30 weeks are shown in Figure 1G.

### Astrocytosis

**Figure 2:**
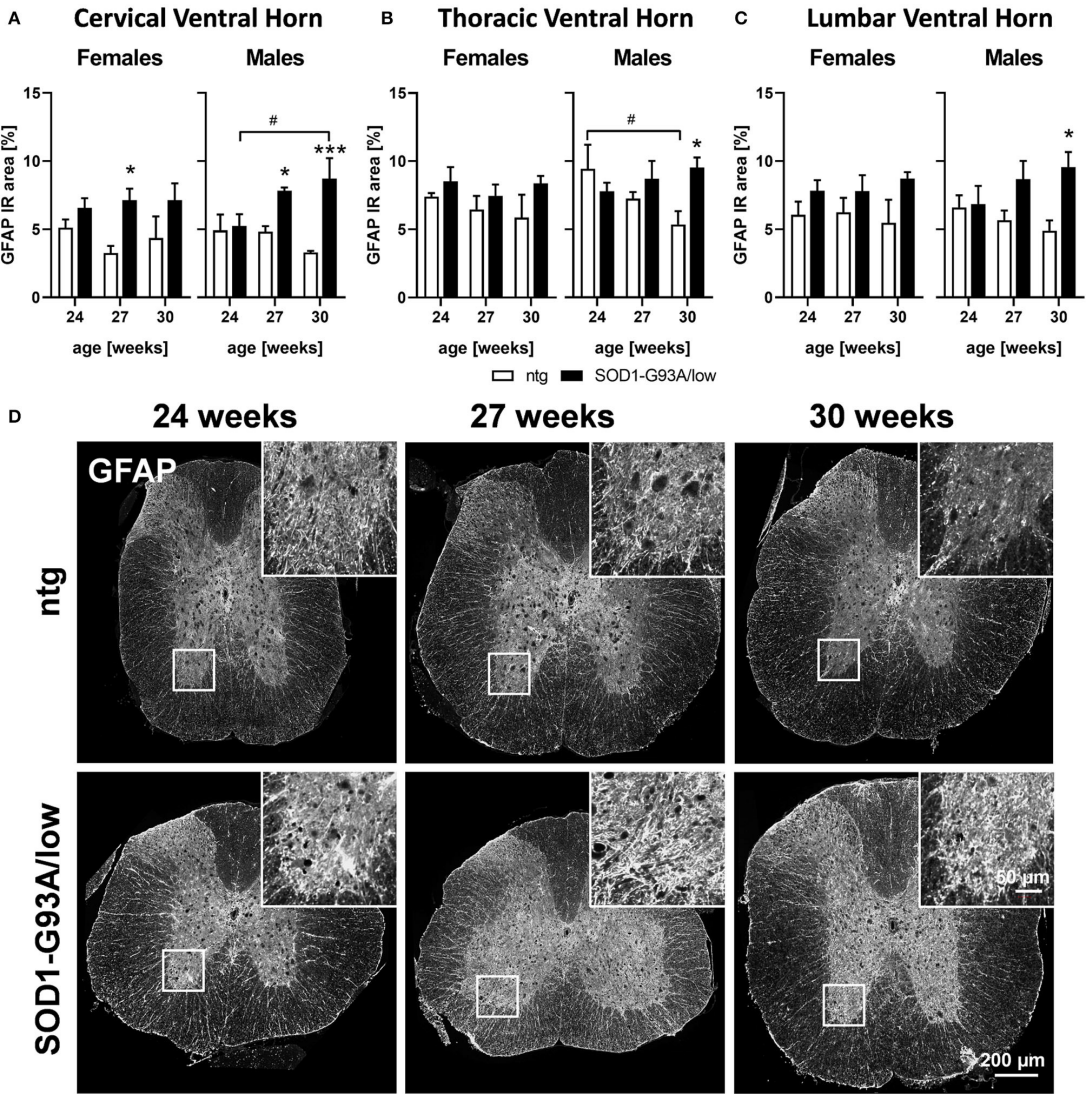
Astrocytosis in the spinal cord of 24–30 weeks old SOD1-G93A/low mice. Female and male 24, 27, and 30 weeks old SOD1-G93A/low mice were evaluated for glial fibrillary acidic protein (GFAP) expression **(A–C)** in the cervical **(A)**, thoracic **(B)**, and lumbar **(C)** ventral horn compared to ntg littermates. Two-way ANOVA with Tukey's and Sidak's multiple comparison *post hoc* test; *n* = 4 per group. Mean + SEM. */^#^*p* < 0.05, ***p < 0.001. *Differences between genotypes; ^#^differences between age groups. **(D)** Representative images of GFAP labeling in the thoracic spinal cord of 24, 27, and 30 weeks old male SOD1-G93A/low mice compared to ntg littermates.

In the next step, we evaluated astrocytosis in the spinal cord of 24, 27, and 30 weeks old female and male SOD1-G93A/low mice compared to ntg littermates. The cervical, thoracic, and lumbar ventral horn was immunofluorescently labeled with a GFAP antibody and the signal was quantified as IR area in percent. The results show that the GFAP IR area was significantly increased in all three regions of male SOD1-G93A/low mice at the age of 30 weeks compared to ntg littermates (Figures 2A–C). In the cervical ventral horn, this difference was already significant at the age of 27 weeks (Figure 2A). While the GFAP IR area constantly increased with age in male SOD1-G93A/low mice, specifically in the cervical ventral horn, the GFAP IR area in female SOD1-G93A/low mice barely changed compared to ntg littermates or with age (Figures 2A–C). Furthermore, the GFAP IR area in all three ventral horn regions of ntg littermates seemed to decrease with age, specifically in the thoracic ventral horn (Figures 2A–C). Representative images of GFAP labeling in the thoracic spinal cord of SOD1-G93A/low mice and ntg littermates at the age of 24, 27, and 30 weeks are shown in Figure 2D.

### Activated Microglia

**Figure 3:**
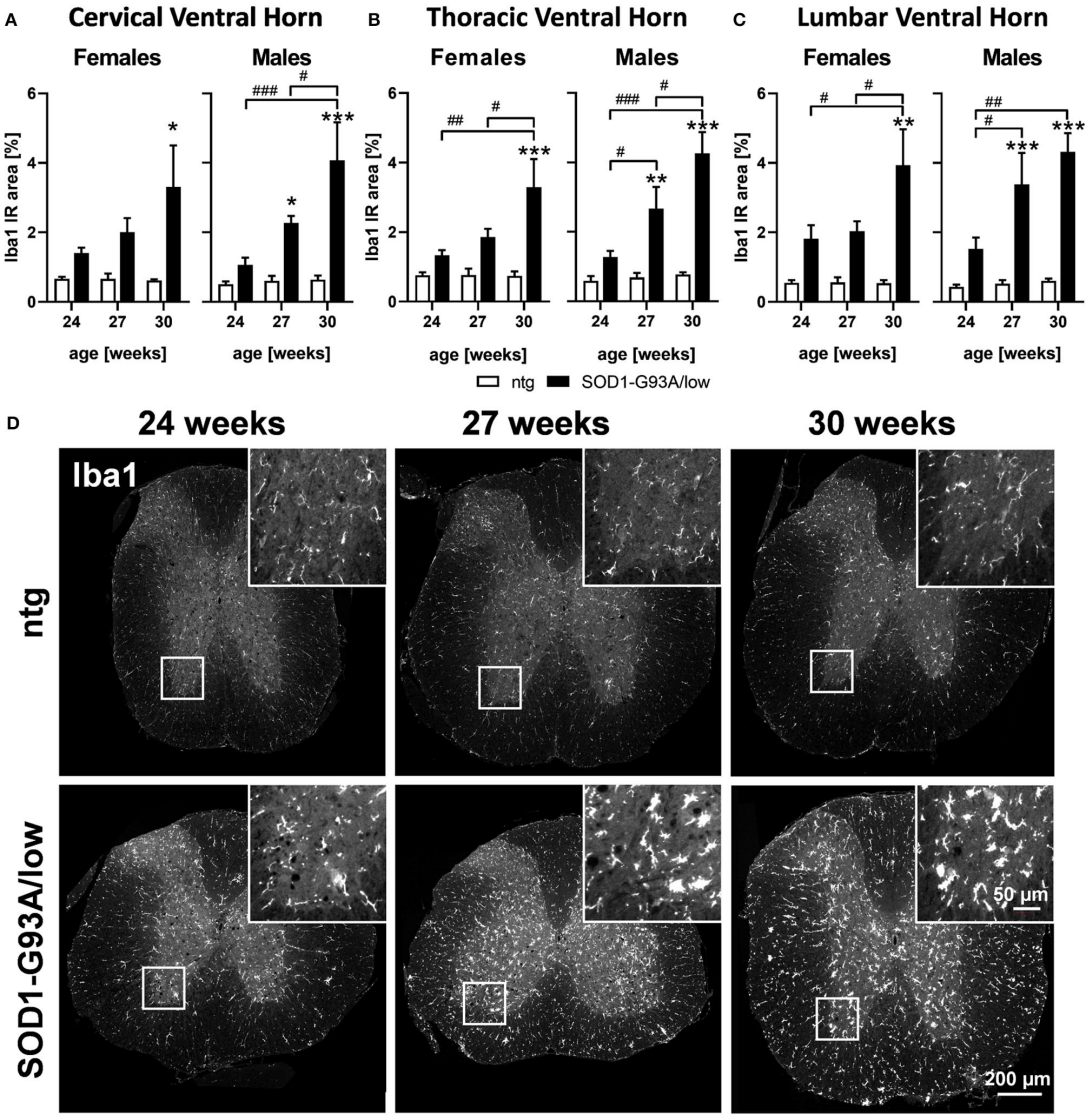
Microgliosis in the spinal cord of 24–30 weeks old SOD1-G93A/low mice. Female and male 24, 27, and 30 weeks old SOD1-G93A/low mice were evaluated for ionized calcium-binding adaptor molecule 1 (Iba1) expression **(A–C)** in the cervical **(A)**, thoracic **(B)**, and lumbar **(C)** ventral horn compared to ntg littermates. Two-way ANOVA with Tukey's and Sidak's multiple comparison *post hoc* test; *n* = 4 per group. Mean + SEM. */^#^*p* < 0.05, **/^*##*^*p* < 0.01, ***/^*###*^*p* < 0.001. *Differences between genotypes; ^#^differences between age groups. **(D)** Representative images of Iba1 labeling in the thoracic spinal cord of 24, 27, and 30 weeks old male SOD1-G93A/low mice compared to ntg littermates.

Evaluation of activated microglia in SOD1-G93A/low mice showed a progressively increasing Iba1 IR area from 24 to 30 weeks of age in all analyzed ventral horn areas of female and male SOD1-G93A/low mice. Differences observed with age were significant in all ventral horn areas of female and male SOD1-G93A/low mice, except the cervical ventral horn of female SOD1-G93A/low mice (Figure 3A). At the age of 30 weeks, the Iba1 IR area of female SOD1-G93A/low mice was increased in all ventral horn areas compared to ntg littermates, while in male animals a significant effect could already be observed at 27 weeks of age. Iba1 IR area of ntg animals stayed very low throughout all age groups (Figures 3A–C).

Representative images of Iba1 labeling in the thoracic spinal cord of SOD1-G93A/low mice and ntg littermates at the age of 24, 27, and 30 weeks are shown in Figure 3D.

### Muscle Phenotype

**Figure 4:**
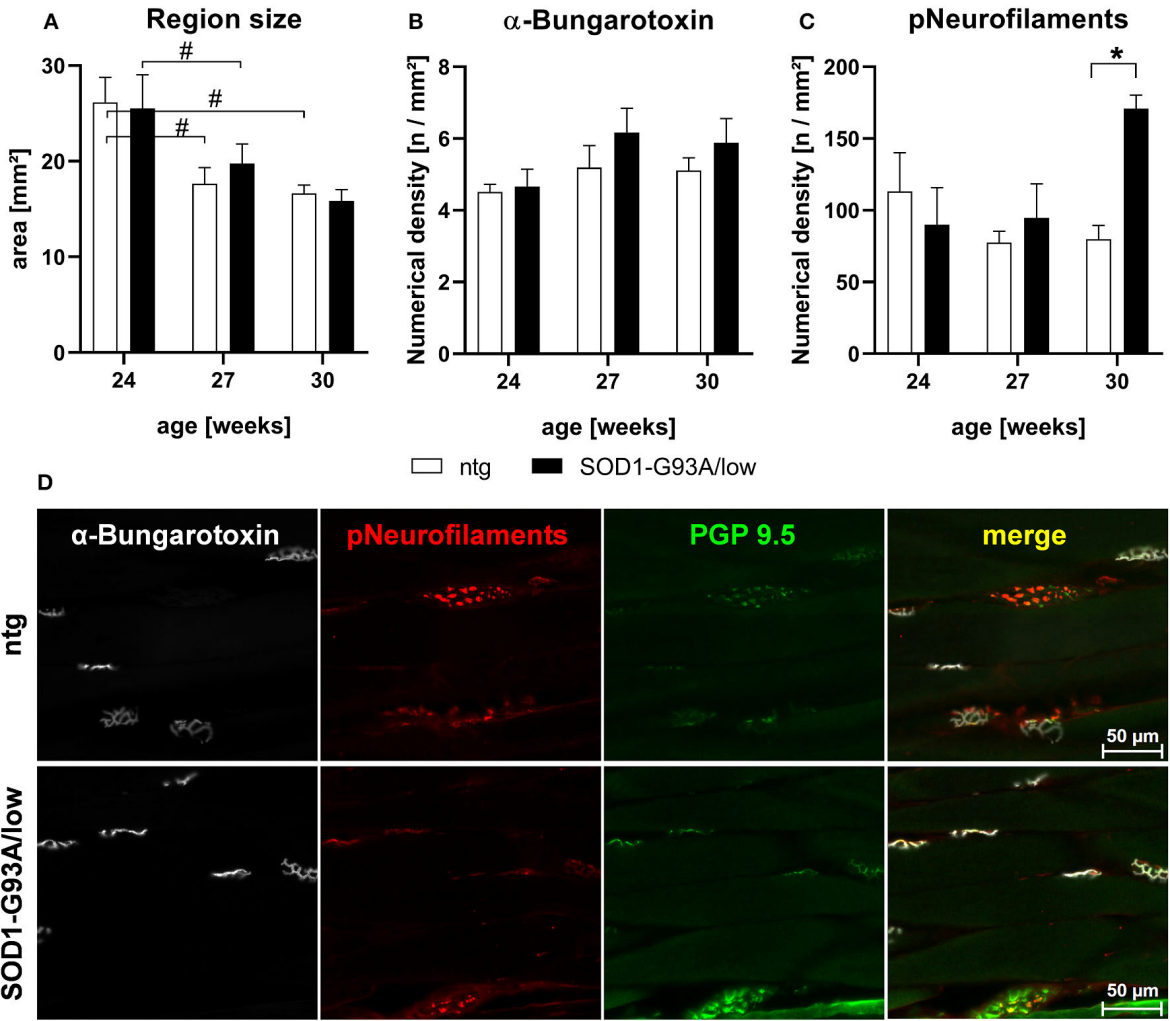
Histological analysis of the musculus gastrocnemius of 24–30 weeks old SOD1-G93A/low mice. The musculus gastrocnemius of 24, 27, and 30 weeks old male SOD1-G93A/low mice was evaluated for region size **(A)**, numerical density of acetylcholine receptors at the postsynaptic surface of neuromuscular junctions by fluorophore-conjugated α-bungarotoxin labeling **(B)**, and numerical density of phosphorylated neurofilaments **(C)** compared to ntg littermates. Two-way ANOVA with Tukey's and Sidak's multiple comparison *post hoc* test; *n* = 4 per group. Mean + SEM. */^#^*p* < 0.05. **(D)** Representative images of α-bungarotoxin (white), phosphorylated neurofilaments (pNeurofilaments, red), and protein gene product (PGP) 9.5 (green) triple labeling in the musculus gastrocnemius of a 30 weeks old male SOD1-G93A/low mouse compared to ntg littermate.

Evaluation of the musculus gastrocnemius of 24–30 weeks old male SOD1-G93A/low mice showed that the size of this skeletal muscle was comparable between SOD1-G93A/low mice and ntg littermates of all age groups. Muscle size significantly decreased with age in SOD1-G93A/low mice and ntg littermates (Figure 4A). Further quantitative analysis of the α-bungarotoxin IR area in the musculus gastrocnemius to label acetylcholine receptors of the postsynaptic surface of neuromuscular junctions (Figure 4B) showed no significant changes in the number of labeled objects between SOD1-G93A/low mice and ntg littermates of all age groups. Quantification of phosphorylated neurofilaments resulted in a significantly increased number of positive objects in 30 weeks old SOD1-G93A/low mice compared to ntg littermates (Figure 4C). Immunofluorescent labeling of α-bungarotoxin and phosphorylated neurofilament was performed in triple labeling together with PGP 9.5, a marker for neuronal fibers. PGP 9.5 labeling could not be quantitatively analyzed, due to a very strong background signal. Representative images of the co-labeling experiment in 30 weeks old male SOD1-G93A/low mouse compared to a male ntg littermate of the same age are shown in Figure 4D.

### General Health and Survival

**Figure 5:**
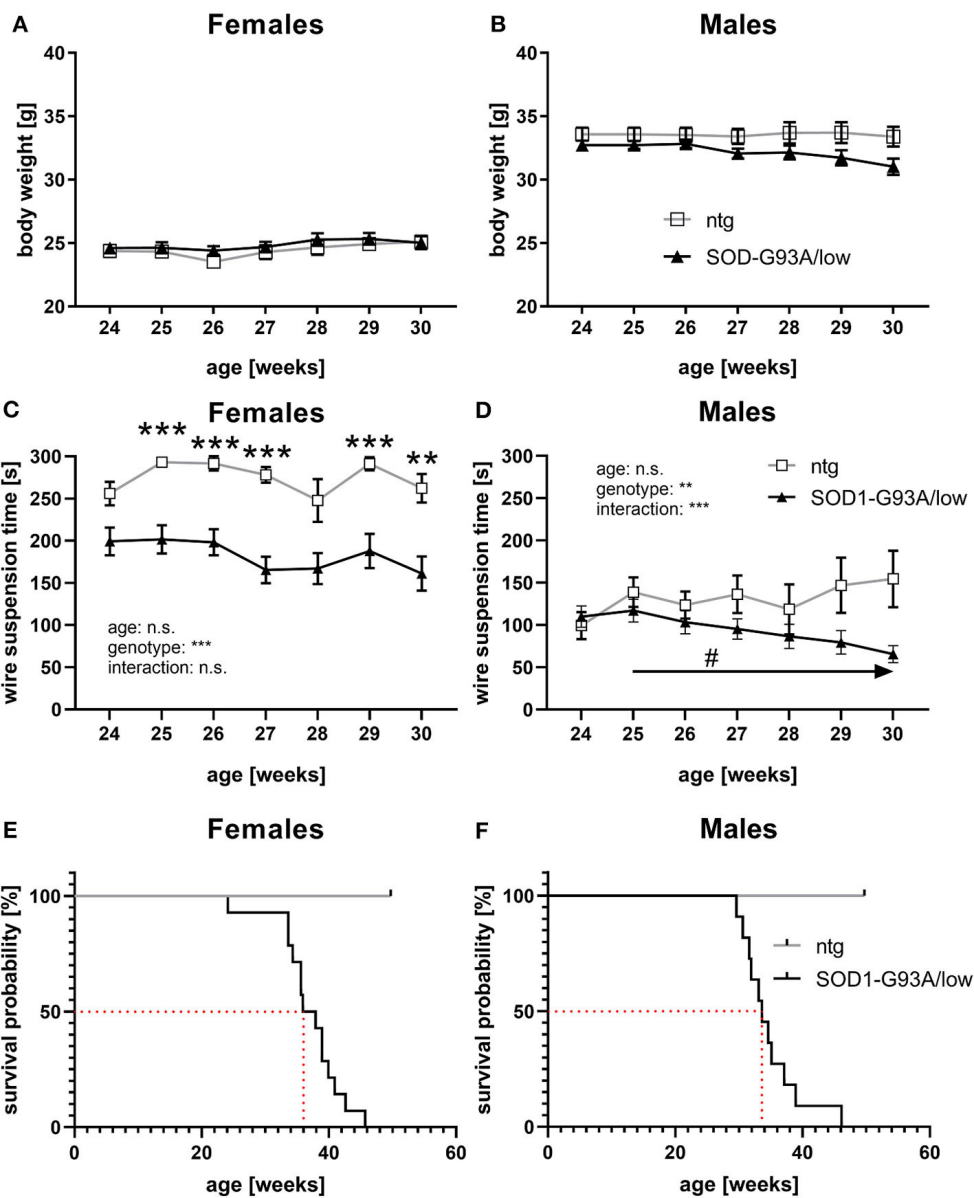
Body weight, muscle strength, and survival rate of SOD1-G93A/low mice. **(A,B)** The body weight of female **(A)** and male **(B)** SOD1-G93A/low mice was evaluated weekly from 24 to 30 weeks. Mean ± SEM. Two-way ANOVA with Tukey's and Sidak's multiple comparison *post hoc* test. All comparisons were not significant. **(C,D)** Mean latency to fall off the wire grid in the wire suspension test of 24–30 weeks old female **(C)** and male **(D)** SOD1-G93A/low mice compared to ntg littermates. Mean ± SEM. Mixed-effects analysis with Tukey's and Sidak's multiple comparison *post hoc* test; ^#^*p* < 0.05, **p < 0.01, ***p < 0.001. *Differences between genotypes; ^#^differences between age groups. **(E,F)** Kaplan-Meier curve showing survival probability of female **(E)** and male **(F)** SOD1-G93A/low mice. Median survival rates are marked with a red dotted line. Group size at start: SOD1-G93A/low: *n* = 39; ntg: *n* = 24.

About 24–30 weeks old female and male SOD1-G93A/low mice were further weekly evaluated for body weight changes. Results show that female SOD1-G93A/low mice had a mean body weight of 24.85 g, while the body weight of ntg mice was very similar to 24.46 g. Body weight values of female SOD1-G93A/low mice and ntg littermates did not change overage (Figure 5A). Evaluation of male SOD1-G93A/low mice revealed a mean body weight of 32.18 g, while the mean body weight of ntg littermates was only slightly higher at 33.56 g. The body weight of male SOD1-G93A/low mice and ntg littermates did also barely change over age (Figure 5B).

Weekly evaluation of the wire suspension time of 24–30 weeks old mice revealed a significantly decreased hanging time of female SOD1-G93A/low mice compared to ntg littermates starting at the age of 25 weeks (Figure 5C). The hanging time of female SOD1-G93A/low mice and ntg littermates barely changed with age (Figure 5C). Evaluation of the wire suspension time of 24–30 weeks old male mice showed a progressive decline of the hanging time of SOD1-G93A/low mice, while the hanging time of ntg littermates did not significantly change (Figure 5D). No differences in the hanging time were observed in male SOD1-G93A/low mice compared to ntg littermates of all age groups (Figure 5D).

During the whole study, dead animals or animals that needed to be euthanized due to reaching a humane endpoint were recorded and the survival rate was evaluated. Female SOD1-G93A/low mice showed a median survival rate of 36.9 weeks, while male SOD1-G93A/low mice survived 33.6 weeks (Figures 5E,F). During the same time, no ntg animal died prematurely or had to be euthanized (Figures 5E,F). All euthanized animals presented severe paralysis, mostly in combination with a reduced righting reflex, reduced general behavior, and dehydration. The necropsy of all animals additionally described several animals with fatty liver, enlarged urinary bladder, and discolored enlarged spleen. Due to the low group size of one male and one female ntg littermate from week 30 onward, statistical analysis for curve comparison of the survival analysis was omitted.

## Discussion

### SOD1 Protein Expression and Brain Atrophy

In the first step, we evaluated SOD1 protein levels in the cervical, thoracic, and lumbar ventral horn of the spinal cord and found already highly increased levels in all analyzed regions in female and male SOD1-G93A/low mice at the age of 24 weeks and a strong additional increase in late-stage male SOD1-G93A/low mice. According to our literature search, no publication previously quantified SOD1 levels in SOD1-G93A/low mice with age. First SOD1 labelings in the original SOD1-G93A mice provide a detailed overview of the localization of SOD1 protein (Gurney et al., 1994; Stieber et al., 2000). Turner et al. (2003) analyzed SOD1 levels with age, showing a strong increase in SOD1 levels within the first 60 days of life in the lumbar spinal cord but not in the cervical spinal cord (Turner et al., 2003). Furthermore, SOD1 levels almost plateaued from the age of 60–120 days. As SOD1-G93A mice present a mean survival time of 129 days, late-stage animals were not analyzed. Unfortunately, the authors of this study do not mention the sex of analyzed animals, so a comparison to the observed strong peak in 30 weeks old SOD1-G93A/low mice in the here presented study is not possible (Turner et al., 2003).

Our results show that only male SOD1-G93A/low mice present minor brain atrophy in the cervical ventral horn, while female animals and other ventral horn regions seem not to be affected. Spooren and Hengerer (2000) could show that SOD1-G93A/low mice have an increased caspase-3 like activity in the spinal cord and that this programmed cell death can be observed mainly in symptomatic animals just before or at the end-stage. Authors conclude that cell death in SOD1-G93A/low mice plays a role in disease progression in the terminal phase (Spooren and Hengerer, 2000). Additionally, in a previous study, we were able to show a progressive increase of neurofilament-light chain levels in the plasma of mixed-sex SOD1-G93A/low mice that were already significantly increased at the age of 24 weeks compared to ntg littermates (Loeffler et al., 2020). As we observed here a median survival rate of 33.6 weeks in male and 36.9 weeks in female SOD1-G93A/low mice, it can be assumed that changes in brain atrophy were not yet measurable by a reduced region size at the time of evaluation.

### Neuroinflammation

We show here for the first time quantifications of the neuroinflammatory markers GFAP and Iba1 in SOD1-G93A/low mice. Previous research on neuroinflammation in SOD1-G93A/low mice already showed GFAP and Iba1 expression in the lumbar spinal cord of 22–34 weeks old male SOD1-G93A/low mice on a C57BL/6J background and thus on a different background as used here. Furthermore, this analysis was performed only qualitatively (Acevedo-Arozena et al., 2011). Two additional studies utilized GFAP and Iba1 expression to evaluate if a mitogen-activated protein kinase or a monoacylglycerol lipase inhibitor alters ALS pathology (Fujisawa et al., 2016; Pasquarelli et al., 2017). While both groups analyzed the lumbar spinal cord, Fujisawa et al. (2016) analyzed male SOD1-G93A/low mice at the age of about 37 weeks and Pasquarelli et al. (2017) used female animals at the age of about 31.5 weeks. Although both groups could observe a treatment effect reducing neuroinflammation, analyses were also performed only qualitatively (Fujisawa et al., 2016; Pasquarelli et al., 2017).

Even though neuroinflammation was previously evaluated qualitatively, here we validate such expression and further provide a qualitative and longitudinal evaluation of astrocytosis and activated microglia by GFAP and Iba1 labeling. Hence, neuroinflammation seems to be a robust phenotype of SOD1-G93A/low mice that can be utilized to evaluate compound effects against ALS pathology.

### Muscle Phenotype

Neurofilaments are currently highly discussed as a biomarker for ALS as levels of neurofilament light-chain are shown to be highly increased in the plasma of patients with ALS (Zetterberg et al., 2007) but also in SOD1-G93A/low mice at the age of 27 weeks (Loeffler et al., 2020). A study by Boylan et al. (2009) was able to show highly increased phosphorylated neurofilament heavy-chain (pNF-H) levels in the blood of patients with ALS but also in SOD1-G93A mice with a high copy number. In this ALS mouse model, authors were even able to show a progressive increase of pNF-H levels (Boylan et al., 2009). Although neurofilaments were measured in the plasma or blood, neurofilaments are known to locate in axons, and to be relevant for axonal transport in healthy neurons and are released into the CSF during axonal damage or even cell death. Sternberger and Sternberger (1983) were able to localize non-phosphorylated neurofilaments in cell bodies, their dendrites, and at least proximal axons, while pNF was found in long fibers, including terminal axons of wild-type rats (Sternberger and Sternberger, 1983). Results of our study even suggest that pNF levels are increased in axons of the neuromuscular junction of 30 weeks old male SOD1-G93A/low mice. Parallel quantification of acetylcholine receptors at the postsynaptic surface of neuromuscular junctions showed no changes between groups, suggesting that the number of motor units of male SOD1-G93A/low mice does not change up to the age of 30 weeks. This result contradicts earlier analyses by Acevedo-Arozena et al. (2011) showing a reduced number of motor units by electrophysiological analysis of the soleus and extensor digitorum longus (EDL) muscle of SOD1-G93A/low mice at the age of 34 and 24 weeks, respectively (Acevedo-Arozena et al., 2011). Although, it needs to be mentioned that analyzed SOD1-G93A/low mice were bred on a different background, and authors analyzed different skeletal muscles compared to the analyzed musculus gastrocnemicus of the here presented study. It can thus not be excluded, that a corresponding phenotype might be observable in older SOD1-G93A/low mice. In accordance with these results is the unaltered muscle size of up to 30 weeks old SOD1-G93A/low mice as a reduced muscle innervation would result in reduced muscle size.

### General Health and Survival

Evaluation of general health and motor deficits in SOD1-G93A/low mice in previous studies showed a symptom onset at about 28.5 weeks of age (Reinholz et al., 1999; Pasquarelli et al., 2017). These groups evaluated body weight, neurological symptoms, like tremor and splay, muscle weakness by gait analysis, and motor deficits by running wheel activity. Although first symptoms were observed before the age of 30 weeks, most parameters were altered later, at the age of about 31–33 weeks. In this study, we evaluated the body weight of SOD1-G93A/low mice from week 24–30 as group sizes were afterward too small. We did not observe major differences in the body weight compared to ntg littermates, neither in female nor in male animals. This result contradicts previous analyses, but can probably be explained by the fact, that animals in the here presented study received food pellets on the cage floor as soon as animals were unable to reach the food grid. This easily accessible food prevented animals from starvation and resulting body weight loss. As the survival rate of SOD1-G93A/low mice of our study was comparable to previous studies (Reinholz et al., 1999; Fujisawa et al., 2016; Pasquarelli et al., 2017; Osman et al., 2020), it can be assumed that the survival rate of SOD1-G93A/low mice was not affected by a reduced food intake in these studies. We further evaluated the muscle strength of SOD1-G93A/low mice using the wire suspension test in 24–30 weeks old animals and found a significantly reduced muscle strength in female SOD1-G93A/low mice already at the age of 25 weeks and thus more than 3 weeks earlier compared to all other so far performed analyses that showed first weaknesses at the age of about 31.5 weeks or later (Reinholz et al., 1999; Pasquarelli et al., 2017). Evaluation of male SOD1-G93A/low mice revealed a much fainter muscle weakness compared to ntg littermates. Observed differences in muscle strength between female and male animals are most likely based on sex-dependent weight differences. Acevedo-Arozena et al. (2011) performed an in-depth analysis of general health, tremor, and muscle strength in SOD1-G93A/low mice. As this analysis was performed in SOD1-G93A/low mice with a C57BL/6J background, a comparison with the shown data is not possible (Acevedo-Arozena et al., 2011).

Our *in vivo* analyses thus suggest that evaluation of the body weight is not a useful parameter to measure general health, while the wire suspension test is a good tool to evaluate early muscle weakness in females and later also in male SOD1-G93A/low mice.

The survival analysis nicely demonstrates the stability of this phenotype in SOD1-G93A/low mice, as similar survival rates were already described by several other groups (Reinholz et al., 1999; Fujisawa et al., 2016; Pasquarelli et al., 2017; Osman et al., 2020). Osman et al. (2020) just recently were even able to show the same difference in the survival rate between female and male SOD1-G93A/low mice, with a longer survival rate of female mice (Osman et al., 2020). As we observed a strong peak in SOD1 protein expression in 30 weeks old male but not female SOD1-G93A/low mice, it is likely that this peak causes the small sex difference in the median survival rate of SOD1-G93A/low mice. Different survival rates as described in the literature might be caused by breeding mice on a different genetic background, housing conditions, or altered copy numbers (Dal Canto and Gurney, 1997; Jung et al., 2001; Acevedo-Arozena et al., 2011). The survival analysis in SOD1-G93A/low mice requires evaluating animals for about 10 weeks and thus double the time of the original SOD1-G93A mouse line. As SOD1-G93A/low mice also live about double the time compared to SOD1-G93A mice, this time window should be well feasible for treatment studies.

### Treatment Studies Using SOD1-G93A/low Mice

In the last 20 years, SOD1-G93A/low mice have been repeatedly used to evaluate the efficacy of new compounds against ALS. All studies were able to show a positive effect of the compound on the survival rate of SOD1-G93A/low mice (Reinholz et al., 1999; Jung et al., 2001; Fujisawa et al., 2016; Pasquarelli et al., 2017). Survival of vehicle-treated SOD1-G93A/low control groups was comparable animals of our study, suggesting that the induced stress caused by the treatment regime did not alter the overall survival rate of SOD1-G93A/low mice. Further readouts that were modifiable by the compounds were neurological symptoms, muscle weakness, and motor ability (Reinholz et al., 1999; Pasquarelli et al., 2017), as well as neuroinflammation by labeling of astrocytes and activated microglia (Fujisawa et al., 2016; Pasquarelli et al., 2017). Our evaluation of SOD1-G93A/low mice further encourages the use of these readouts for future efficacy studies. Based on the presented results, we suggest a treatment start at the age of 24 weeks or younger when treatment needs to be started before pathology onset, while treatment would need to be started at the age of 27 weeks or later when treatment needs to be started after pathology onset.

## Conclusion

In summary, we show pathological features of 24–30 weeks old female and male SOD1-G93A/low mice. Our data proof a stronger neuroinflammatory pathology of male SOD1-G93A/low mice that are accompanied by very high SOD1 protein levels in the spinal cord and a reduced survival rate. Our data thus suggest that specifically male SOD1-G93A/low mice are a great model to evaluate the efficacy of new drugs against ALS, as most analyzed pathologies develop progressively and thus allow enough time for pharmacological intervention.

## Data Availability Statement

The original contributions presented in the study are included in the article/Supplementary Material, further inquiries can be directed to the corresponding author/s.

## Ethics Statement

The animal study was reviewed and approved by Amt der Steiermärkischen Landesregierung, Abteilung 13 – Umwelt und Raumordnung Austria.

## Author Contributions

AM-K designed, performed, analyzed *in vivo* tests, behavioral experiments, and edited the manuscript. BH designed, performed, analyzed histological labelings, prepared corresponding figures, and wrote and edited the manuscript. RR planned and designed *in vivo* tests, behavioral analyses, and edited the manuscript. JN designed and analyzed histological labelings and edited the manuscript. SF prepared figures, interpreted results, and wrote and edited the manuscript. BH-P conceived the study, interpreted the results, and edited the manuscript. All authors contributed to the article and approved the submitted version.

## Conflict of Interest

All authors are employed by the company QPS Austria GmbH. The authors declare that this study received funding from QPS Austria GmbH. The funder was not involved in the study design, collection, analysis, interpretation of data, the writing of this article or the decision to submit it for publication.

## Abbreviations

AAALAC: Association for Assessment and Accreditation of Laboratory Animal Care
ALS: Amyotrophic Lateral Sclerosis
CNS: the central nervous system
GFAP: glial fibrillary acidic protein
Iba1: ionized calcium-binding adaptor molecule 1
IR: immunoreactive
ntg: non-transgenic
PGP 9.5: Protein Gene Product 9.5
SOD1: superoxide dismutase 1 gene.
